# Retraction Note: Probiotic assisted weight management as a main factor for glycemic control in patients with type 2 diabetes: a randomized controlled trial

**DOI:** 10.1186/s13098-023-01094-x

**Published:** 2023-05-26

**Authors:** Leila Khalili, Beitullah Alipour, Mohammad Asghari Jafarabadi, Tohid Hassanalilou, Mehran Mesgari Abbasi, Ismail Faraji

**Affiliations:** 1 Department of Nutrition, Faculty of Nutrition and Food Sciences, Tabriz University of Medical Sciences, Tabriz, Islamic Republic of Iran; 2 Department of Community Nutrition, Faculty of Nutrition and Food Sciences, Tabriz University of Medical Sciences, Tabriz, Islamic Republic of Iran; 3 Department of Statistics and Epidemiology, Faculty of Health, Road Trafc Injury Research Center, Tabriz University of Medical Sciences, Tabriz, Islamic Republic of Iran; 4 Drug Applied Research Center, Tabriz University of Medical Sciences, Tabriz, Islamic Republic of Iran; 5 Internist, Fellow of Endocrinology, Tabriz University of Medical Sciences, Tabriz, Islamic Republic of Iran

Retraction: Khalili *et al. Diabetol Metab Syndr (2019) 11:5*

10.1186/s13098-019-0400-7

The Editor in Chief has retracted this article because it has been previously published by some of the same authors [1]. This article is therefore redundant. None of the authors have responded to any correspondence from the editor or publisher about this retraction.
